# Quantifying multiple stain distributions in bioimaging by hyperspectral X-ray tomography

**DOI:** 10.1038/s41598-022-23592-0

**Published:** 2022-12-19

**Authors:** Ryan Warr, Stephan Handschuh, Martin Glösmann, Robert J. Cernik, Philip J. Withers

**Affiliations:** 1 Henry Royce Institute, Department of Materials, The University of Manchester, Manchester, M13 9PL UK; 2 VetCore Facility for Research, University of Veterinary Medicine Vienna, Vienna, Austria

**Keywords:** 3-D reconstruction, X-ray tomography, Biomarkers, Tissues, Characterization and analytical techniques

## Abstract

Chemical staining of biological specimens is commonly utilised to boost contrast in soft tissue structures, but unambiguous identification of staining location and distribution is difficult without confirmation of the elemental signature, especially for chemicals of similar density contrast. Hyperspectral X-ray computed tomography (XCT) enables the non-destructive identification, segmentation and mapping of elemental composition within a sample. With the availability of hundreds of narrow, high resolution (~ 1 keV) energy channels, the technique allows the simultaneous detection of multiple contrast agents across different tissue structures. Here we describe a hyperspectral imaging routine for distinguishing multiple chemical agents, regardless of contrast similarity. Using a set of elemental calibration phantoms, we perform a first instance of direct stain concentration measurement using spectral absorption edge markers. Applied to a set of double- and triple-stained biological specimens, the study analyses the extent of stain overlap and uptake regions for commonly used contrast markers. An improved understanding of stain concentration as a function of position, and the interaction between multiple stains, would help inform future studies on multi-staining procedures, as well as enable future exploration of heavy metal uptake across medical, agricultural and ecological fields.

## Introduction

**Figure 1:**
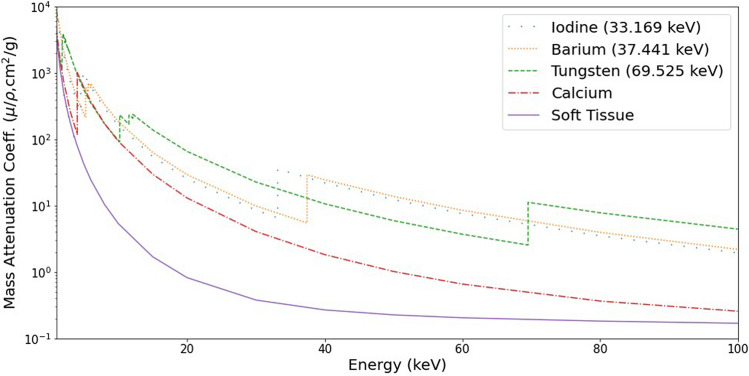
Attenuation variation as a function of energy. Known values of the mass attenuation coefficient shown for a number of materials. Across the hard X-ray energy range, many elements exhibit sharp rises in attenuation at their absorption edge positions (K-edge energies given in legend), providing strong contrast relative to soft tissue or bone (calcium) structures. Values extracted from the NIST online database^2^.

X-ray computed tomography (XCT) has long been used to analyse the internal structure and composition of samples non-invasively across a wide range of fields, including the study of biological soft tissue structures. Compared to destructive techniques, such as microscopic histology, the use of XCT enables whole samples to be examined in 3D in a single scan, to obtain insight on the internal organisation of the full specimen. Achieving strong contrast is a key aspect of XCT in order to confidently resolve structures and segment distinct regions. Contrast between material phases is dependent on their relative absorption (attenuation) of X-rays. Every element and material substance has a unique, energy-dependent attenuation profile, with materials of higher density such as bone (calcium) more readily absorbing of X-rays than say, unmineralised (soft) tissue. Some examples of attenuation profiles for chemical elements, as well as that of soft tissue, are shown in Fig. 1. With increased X-ray energy, differences in attenuation narrow, and therefore contrast is reduced. As such, for the hard X-ray energy range (> 20 keV), feature separation can be difficult, particularly for soft tissue imaging of biological samples with XCT^1^.

One method of improved tissue characterisation is through the addition of contrast agents. These chemical stains, composed of highly-attenuating elements such as iodine or tungsten, offer a form of localised contrast enhancement due to their differing affinities to soft tissue structures, increasing tissue attenuation. Popular stains include the use of iodine-based compounds (for deep and rapid staining of multiple tissues^3–6^) as well as phosphomolybdic and phosphotungstic acid (PMA, PTA) for their slower, but strong binding to proteins and connective tissue^1,7,8^.

XCT enables the imaging of stained soft tissues at high contrast, however it cannot readily discriminate two or more highly-absorbing materials present in the same sample. Material differentiation can instead be accomplished by the evaluation of attenuation values at multiple energies. Common X-ray contrast agents display sharp discontinuities in X-ray attenuation at specific X-ray energies according to their elemental composition. The sharp jumps in attenuation, as observed in Fig. 1, correspond to K-edges, occurring at the binding energy of an element’s K-shell electrons. By measuring the attenuation at a number of energies, the aforementioned differences in absorption for soft tissues may be exploited to discriminate between them. Dual-energy CT (DECT) utilises this comparison of relative attenuation by exposing the tissues to X-ray spectra at two different energies. Multiple DECT geometries have been created, such as rapid voltage-switching, dual source systems or even dual-layer detector technologies^9^, however the principle is still the same. By evaluating the attenuation response of the sample at two different X-ray energies, any differences in relative attenuation may be exposed and used as a means of material discrimination^10^. If a contrast agent is also added, then by selecting X-ray spectra at energies before and after the K-edge value, attenuation differences between stained and unstained regions are exaggerated, improving soft tissue identification. Biological applications of DECT have included imaging of vasculature^11^, gout^12^ and atherosclerotic plaque^13,14^. The method of DECT is disadvantaged however by the increased dose required^15^, as well as the difficulty in unambiguously differentiating spectrally similar chemicals, such as that of barium and iodine contrast agents.

Developments in single photon-counting detector systems have aimed to bridge the gap between conventional absorption X-ray CT/DECT and full chemical characterisation modalities, by enabling the simultaneous acquisition and measurement of photon energy and position at each respective pixel. A key advantage to such systems is the ability to directly capture spectral markers, such as K-edges and X-ray fluorescence (XRF) peaks. The creation of detector systems with high spectral sensitivity over a very narrow bandwidth (≤ 1 keV), termed ‘hyperspectral’ detectors, have enabled full access to these markers, with the energy precision required to unambiguously match them to their elemental origins. With imaging possible over the hard X-ray range, these systems are also well-suited for lab-based CT imaging, where a polychromatic spectrum may be captured in full, and recorded over hundreds of energy channels. As such, hyperspectral imaging facilitates the simultaneous recording, and mapping, of a wide range of chemical elements, to provide complete 4D spatiospectral insight. Previous applications of hyperspectral CT have explored elemental segmentation with K-edges in geology^16^ and single-stain bioimaging^17^, XRF imaging of nanoparticles^18^ and alloy melting/solidification^19^, as well as spectral diffraction studies of brain amyloid plaque^20^.

As shown elsewhere^16,17^, the size of an absorption edge may be measured to determine relative chemical concentration as a function of spatial position in the sample. However, with the addition of a suitable calibration phantom, it is possible to precisely extract the direct relationship between absorption step size and chemical concentration, to produce maps of absolute concentration. As such, for multiply-stained biological specimens, hyperspectral imaging provides a unique ability to further understand and quantify the interaction and distribution of simultaneous contrast agents over a variety of soft tissue regions.

Testing popular and commonly used contrast agents, here we evaluate the ability to segment similarly-attenuating chemicals, and measure their concentration distribution across skin, muscle, connective tissue and vasculature in murine specimens. In order to investigate the efficacy of hyperspectral tomography for multi-stain bioimaging we examine a mouse hindlimb stained with two contrast agents (barium and iodine) and a mouse forelimb stained with three (barium, iodine and tungsten). In addition, we establish a calibration routine using a set of idealised phantoms of the same chemical stains. The spectroscopic imaging routine, here using the hyperspectral HEXITEC detector system, is verified against results achieved in conventional XCT and DECT, providing a foundation for automated elemental concentration measurement across a range of samples and research fields in the future. The unique ability to directly identify, quantify and map heavy elements shown in this study demonstrates the powerful potential of hyperspectral X-ray CT as a tool for non-destructive, 3D chemical analysis, applicable to research including medical biopsies and heavy metal poisoning.

## Methods

As detailed below, all methods and procedures are reported in accordance with the ARRIVE guidelines, and the relevant legislation.

### Biological sample and phantom preparation

All procedures followed animal care guidelines approved by the Administrative Panel on Laboratory Animal Care of the University of Veterinary Medicine in Vienna. No experimental procedures were carried out on living animals. Tissues for contrast-staining were collected from a single male, adult C57BL/6 N mouse. The animal was specific pathogen-free (SPF) according to FELASA recommendations^21^ and maintained in a barrier rodent facility. For mice that are not genetically modified (such as C57BL/6 N), breeding as well as euthanasia for the mere purpose of tissue harvesting is not subject to permission by license according to national legislation of Austria and EU directive 2010/63/EU. Inhalation euthanasia was performed by an overdose of Isoflurane in accordance with international standards and recommendations for humane euthanasia^22,23^. The mouse was placed in a closed receptacle containing gauze soaked with 5 ml Isoflurane. Inside the receptacle, a barrier of dry paper tissue ensured that the animal was only exposed to anaesthetic vapour and did not get in contact with the liquid Isoflurane. The animal was kept in the closed receptacle for ten minutes. Cessation of heartbeat and breathing occurred within five minutes. After ten minutes, the mouse was taken out of the receptacle and successful euthanasia was verified by the absence of heartbeat, breathing and toe pinch reflex. All perfusion and tissue preparation procedures were carried out post-mortem and thus also were not subject to permission by license according to national legislation of Austria and EU directive 2010/63/EU.

### Mouse hindlimb containing two high-Z contrast agents (barium, iodine)

The mouse was perfused into the left ventricle with isotonic saline containing 10 UI/ml Heparin to remove blood from the vasculature, followed by perfusion fixation with 4% neutral buffered formalin. Subsequently, the vasculature was perfused with 30% Micropaque® barium sulphate (w/v) in 3% gelatine in phosphate buffered saline (PBS). After perfusion, the whole mouse was immersed in 4% neutral buffered formalin at 4 °C for 72 h. After fixation, the head, limbs and inner organs were harvested and stored in PBS at 4 °C. A hindlimb was dehydrated to absolute ethanol through a graded series of ethanol concentrations (70, 85, 95, 100%, 1 h per step). The dehydrated hindlimb was stained using 1% elemental iodine (I_2_) in absolute ethanol for six days at room temperature (RT) using gentle horizontal shaking. After staining, the hindlimb was washed in absolute ethanol and mounted in a polypropylene tube again in absolute ethanol.

### Mouse forelimb containing three high-Z contrast agents (barium, iodine, tungsten)

Details on fixation and perfusion have been reported above. A forelimb was transferred to 70% ethanol. In the first staining step, the forelimb was immersed in 1% (w/v) phosphotungstic acid hydrate (PTA) in 70% ethanol for 11 days at RT using gentle horizontal shaking. Subsequently, the sample was transferred to an acidic (pH 2.8, pH adapted using HCl) low concentration I_2_KI solution containing 0.25% (w/v) I_2_ and 0.5% (w/v) KI in distilled water and stained for six days at RT using gentle horizontal shaking. Finally, the forelimb was mounted in the same acidic (pH 2.8) low concentration I_2_KI solution inside a polypropylene tube. Staining times used for the sample enable a complete staining from the cut surface to the elbow including all soft tissues of the upper arm, which was the main ROI in the present investigation. However, a longer PTA staining time would be required to completely stain soft tissues of the lower arm and paw.

### BaSO_4_ phantom

Three mixtures of commercial Micropaque® barium sulphate suspension (1 g BaSO_4_/ml) with 2% agarose gel were made. First, the agarose was heated to 70 °C. Then, the respective amount of BaSO_4_ and agarose was mixed with a Vortex Mixer, and pipetted into small plastic containers, followed by immediate cooling to avoid sinking of the BaSO_4_ crystals. In total three concentrations of BaSO_4_ were prepared, equating to 100, 200, and 400 mg/ml, respectively. The three plastic containers were put into a 5 ml polypropylene tube, and the space around the three containers was filled with 2% agarose that solidified at RT.

### Iodine phantom

Four containers of aqueous I_2_KI were prepared, with concentrations of 25.3, 50.6, 76.0 and 101.2 mg of I_3_^-^ per ml respectively. The containers were held in an interchangeable setup to enable the locations of each concentration to be switched as required.

### PTA phantom

Three concentrations of aqueous PTA solutions were prepared, containing 50, 100, and 200 mg of phosphotungstic acid hydrate per ml. The three plastic containers were put into a 5 ml polypropylene tube, and the space around the three containers was filled with 2% agarose that solidified at RT.

### Hyperspectral X-ray microCT imaging

All samples were scanned with a 225 kV Nikon X-ray source, based within the High Flux walk-in bay at the Henry Moseley X-ray Imaging Facility (HMXIF), University of Manchester. The experimental set-up for hyperspectral image acquisition follows that of a conventional system. The cone-beam source, sample rotation stage and detector are oriented in a parallel configuration along the same imaging plane. The key difference is that here we replace the standard energy-integrating detector with an energy-sensitive, hyperspectral imaging detector. In this case we use the high-energy HEXITEC hyperspectral detector^24,25^. The HEXITEC detector is capable of measuring both incident photon position and energy, to build an attenuation profile across hundreds of narrow energy channels in each pixel. The result is that any single radiograph may be viewed at a single energy channel, directly exposing changes in attenuation as a function of energy. The HEXITEC consists of an 80 × 80 pixel array at a 250 µm pixel pitch, with a 1 mm thick CdTe sensor bump-bonded to an ASIC to produce a 2 cm × 2 cm detection area. The detector has an achievable energy resolution of 800 eV at 60 kV and < 1.5 keV up to 160 kV. Due to the small FOV, all samples investigated in the study were chosen such that all key features could be seen in a single CT acquisition. In addition, the samples only contained features with sizes of ≥ 100 µm, given the relatively poor spatial resolution of the HEXITEC. Prior to scanning of the samples, calibration of the detector was performed by acquiring fluorescent X-ray signals from a series of metal foils, emitted upon exposure to a radioactive ^241^Am source. Matching of the theoretical X-ray fluorescence peak energies to their measured energy channel position enabled a linear fitting to be determined, providing a direct energy-to-channel conversion^26^ (see Supplementary Fig. S1). The energy resolution of the system was determined based on the FWHM of the ^241^Am photopeak at 59.5 keV, averaged over every pixel^17,24^, with resolution measured as 1.27 ± 0.47 keV (see Supplementary Fig. S2). An inter-pixel gain correction was also applied to account for differences in spectral response across the pixel array^27^.

For each sample, the X-ray settings were chosen to strike a balance between measuring the spectral signal from each distinct chemical stain, while also ensuring strong image contrast was obtained. As such, the mean X-ray energy was selected to be high enough to capture a significant number of photons at each known chemical absorption edge. Table 1 shows the selected scan settings for each of the biological samples, as well as the individual chemical phantoms.

**Table 1:** Sample preparation and scan settings.

Sample	Stains	Beam voltage (kV)	Beam current (µA)	Exposure time (s)	Projections	Voxel size (µm)
Mouse hindlimb	I_2_/BaSO_4_	60	13	120	180	151
Mouse forelimb	I_2_KI/BaSO_4_/PTA	90	13	120	180	128
Phantom 1	I_2_KI	60	13	120	180	223
Phantom 2	BaSO_4_	60	13	120	180	196
Phantom 3	PTA	90	20	120	180	177

A full list of the different contrast agents and scan settings for each biological sample and phantom examined in this paper.

### Raw data processing

For each sample, additional processing of the raw data was applied following creation of the 4D sinogram. A common artefact observed following CT reconstruction is that of ring artefacts, due to poorly functioning or miscalibrated detector pixels. Applying the method developed by Münch et al.^28^, which uses a combined wavelet-Fourier based approach, reduces the presence of such artefacts in the final reconstructed volume. In addition, a centre-of-rotation correction was applied to sample datasets where there existed a misalignment from the true centre during scanning. Pre-processing was performed using scripts in MATLAB.

### High resolution XCT/DECT imaging

For the mouse hindlimb and forelimb, respective XCT and DECT scans were performed to provide high spatial resolution comparative datasets to those acquired with hyperspectral imaging. For the hindlimb, the XCT scan used a MicroXCT-400 (Carl Zeiss X-Ray Microscopy, Pleasanton, CA, USA), with a 0.4 × detector assembly, at source settings of 80 kVp, 100 µA. The reconstructed voxel size was 13.63 µm. The forelimb was scanned using the same system and 0.4 × detector assembly, but as a dual-energy set-up. Two scans were acquired at 40 kVp/200 µA and 80 kVp/100 µA, with a reconstructed voxel size of 7.97 µm. Three material fractions were calculated using a post-reconstruction basis material decomposition approach, as described elsewhere^10^. Joint dual energy profiles of bone (hydroxyapatite) and iodine-stained soft tissue were measured using ROIs in the registered dual energy data. Using an energy pair of 40kVp/80kVp, bone and PTA, as well as iodine and barium, show almost identical dual energy profiles. This is because the mean energy of each spectra (~ 27 keV for 40 kVp and ~ 42 keV for 80 kVp) produce similar attenuation around the K-edges of iodine and barium (33.17 and 37.44 keV, respectively), but are both far from the K-edges of calcium and tungsten (4.04 and 69.53 keV, respectively). The result of the basis material decomposition were three image channels (‘materials’): channel 1 = bone + PTA, channel 2 = iodine + barium, channel 3 = water.

### Reconstruction routine

A regularised iterative approach was adopted, using the Primal–Dual Hybrid Gradient (PDHG) algorithm^29^, combined with separate regularising parameters applied to the spatial and spectral dimensions. In this case, we used a method known as TV-TGV regularisation, based on different forms of the Total (Generalised) Variation regularising routine. The algorithm has previously been applied successfully to improve reconstructed image quality for hyperspectral X-ray and neutron tomography^17,30^. Variations of the TV method are known to be efficient at suppressing noise^31,32^, and using a split approach of applying TV and TGV to spatial and spectral domains respectively enabled optimisation for the differing levels of noise in each dimension. Optimisation of weighting parameters plays a crucial role in obtaining high signal-to-noise ratio and for performing precise analysis of the spectral attenuation profiles. Under- or over-estimation of the parameters can lead to little noise suppression or over-smoothing, affecting the identification of weak spectral markers. Therefore, parameters for spatial and spectral regularisation were optimised by trialling a range of values for each dataset, until values were selected to obtain a balance between sufficient noise suppression and strong feature contrast. The reconstruction method was implemented using the open-source Core Imaging Library (CIL) software^33,34^, and fully programmed through Python. All samples were reconstructed with 1000 iterations of the algorithm, which has proven to be sufficient for noise suppression in previous hyperspectral datasets^17^.

### Volume registration

In order to compare exactly corresponding slices between hyperspectral and XCT/DECT datasets, image volumes were imported into the 3D software package Amira 2021.1. (FEI Visualization Sciences Group, part of Thermo Fisher Scientific, Mérignac Cédex, FR). Co-registration of datasets was performed using the *Register Images* tool. The respective XCT/DECT dataset was registered to one energy channel (38.94 keV for double-stained hindlimb, 38.13 keV for triple-stained forelimb) of the hyperspectral data based on normalized mutual information and using a rigid body transformation (six degrees of freedom, translation and rotation in X–Y–Z). After co-registration of image volumes, the corresponding slice was generated with the *Resample Transformed Image* tool using Lanczos interpolation.

### Spectral analysis routines

#### K-edge subtraction

In order to segment and visualise different stained tissue phases, the spectral analysis method of K-edge subtraction (KES) was applied. The method used two key parameters: width and separation. Width determined how many energy channels were integrated either side of the absorption edge, while separation measured the gap between the channel of the known edge position, and the regions either side over which channels are integrated and subtracted. The separation value was determined based on the minimum number of channels needed to span the full absorption edge for each element. A value above zero is often required as experimentally measured absorption edges show a rise in attenuation over a narrow range of channels, compared to the sharp discontinuity observed in theoretical data. For the iodine and barium K-edges in the study, optimal width and separation values of 5 and 2 channels were used, respectively. The separation was increased to 10 channels for the tungsten K-edge in the triple-stained specimen due to a broader absorption edge measured. The method was applied using Python code, producing elemental segmentation maps for each unique K-edge position^16^.

#### Edge height analysis

Following measurement of individual voxel spectra and identification of an absorption edge, the relative size of the absorption edge step was calculated in Python. Linear fits were applied to the attenuation profile either side of the absorption edge, before extrapolating the fits to the known K-edge value. The difference in attenuation value (Δµ_0_) between the upper and lower bounds of the linear fit provide a relative measure of the change in phase attenuation.

#### Absolute concentration calculation

Applying linear fitting to the edge height analysis for the chemical phantoms, the relationship between concentration and step size was determined. The fitted relationship was then used as a baseline for determining absolute concentration of staining agents in the soft tissue structures of each sample. For each sample, the equation corresponding to the linear fit for each contrast agent was used to directly convert edge height values to absolute concentration values. The method was applied iteratively in Python on a voxel-by-voxel basis to produce a 3D map of concentration values for each elemental stain.

## Results

### Elemental segmentation of multi-stain specimens

Our first sample examines a case of double-staining within a mouse hindlimb. The sample was stained with elemental iodine (I_2_) for general soft tissue contrast enhancement, while barium sulphate (BaSO_4_) was added to improve the visualisation of the blood vessels within the limb^35,36^. Despite the relatively poor spatial resolution achievable with the HEXITEC detector it is well suited for imaging the upper knee region of the hindlimb. Indeed, the larger blood vessels in the hindlimb vasculature can reach hundreds of microns in diameter^37^. Here, the use of these two stains represents an example of contrast agents difficult to spectrally separate with DECT due to their similar attenuation profiles and closely-spaced K-edges. A complete breakdown of the scanning parameters, for all biological specimens and phantom samples used in this study, is shown in Table 1. Following scanning, the datasets were reconstructed using an iterative algorithm catered towards optimising image quality for 4D hyperspectral imaging. Given the count-rate limitations of hyperspectral detector systems, a conventional CT reconstruction algorithm such as cone-beam filtered back-projection (FDK) is not well suited, as this algorithm often results in noisy 4D reconstructed volumes, as shown elsewhere^17,30^.

**Figure 2:**
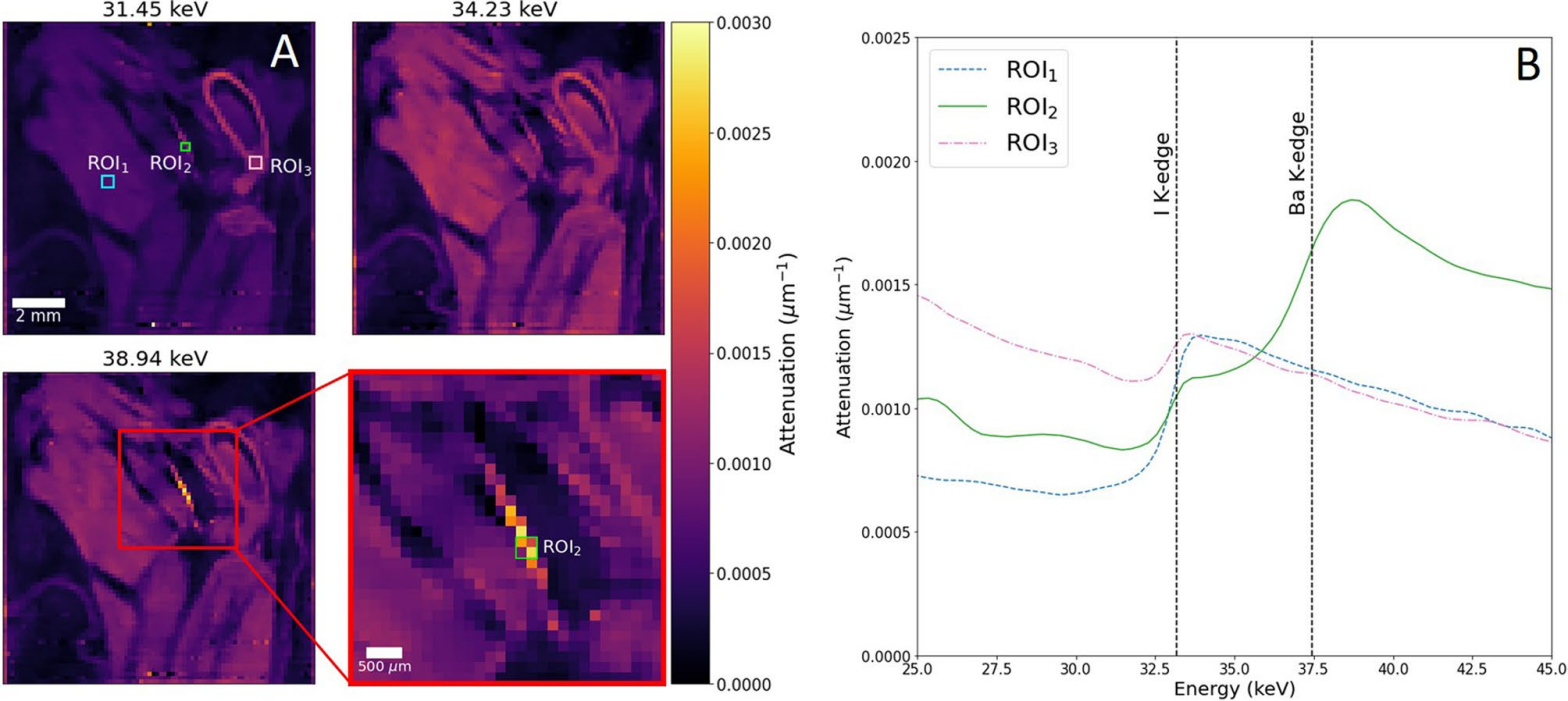
Voxel spectra analysis for double-stained hindlimb specimen. (**A**) Single image slice in the sagittal plane across three monochromatic energy channels, following iterative reconstruction. A set of three regions-of-interest (ROIs) are highlighted for voxel spectra analysis. An enlarged image of a section of (**A**)—red box—is included to highlight ROI_2_ taken over one of the distinct material phases. ROIs_1,3_ cover a 3 × 3 voxel region, while a 2 × 2 pixel region is used for ROI_2_, partially overlapping with surrounding material outside of the phase. (**B**) Voxel spectra for each ROI, showing clear steps in attenuation. Known absorption edge positions (black dotted lines) confirm the presence of iodine and barium in the ROIs, while ROI_3_ shows a small iodine signal, having partially diffused into the calcium-containing bone.

Figure 2A presents a set of reconstructed images corresponding to the same CT slice of the hindlimb, but recorded for three energy channels. A sharp rise in attenuation between the first two energy channels is consistent with the presence of the iodine K-edge (33.17 keV) between them. With approximately 99% of the soft tissue estimated to be stained by iodine, as expected the majority of the reconstructed cross-section shows a consistent attenuation increase. An additional benefit of evaluating quasi-monochromatic image slices is to qualitatively identify different phases, prior to spectral segmentation. The spectral profiles for three regions of interest (ROIs) in Fig. 2A are shown in Fig. 2B. The spectra for ROI_1_ indicates the presence of the iodine contrast agent, with a rise in attenuation at the energy corresponding to the iodine K-edge. Similarly, ROI_2_ includes an absorption step at the energy corresponding to the K-edge of barium, as well as a small rise around the iodine K-edge. The magnified image in Fig. 2A highlights the marginal overlap of ROI_2_ with material outside of the phase, due to the small feature size being analysed, which probably explains the presence of the iodine absorption step. Analysis of ROI_3_, within the dense bone region, shows a minor increase due to the presence of iodine, which has been observed in similar staining studies based on DECT data (SH, personal observation).

**Figure 3:**
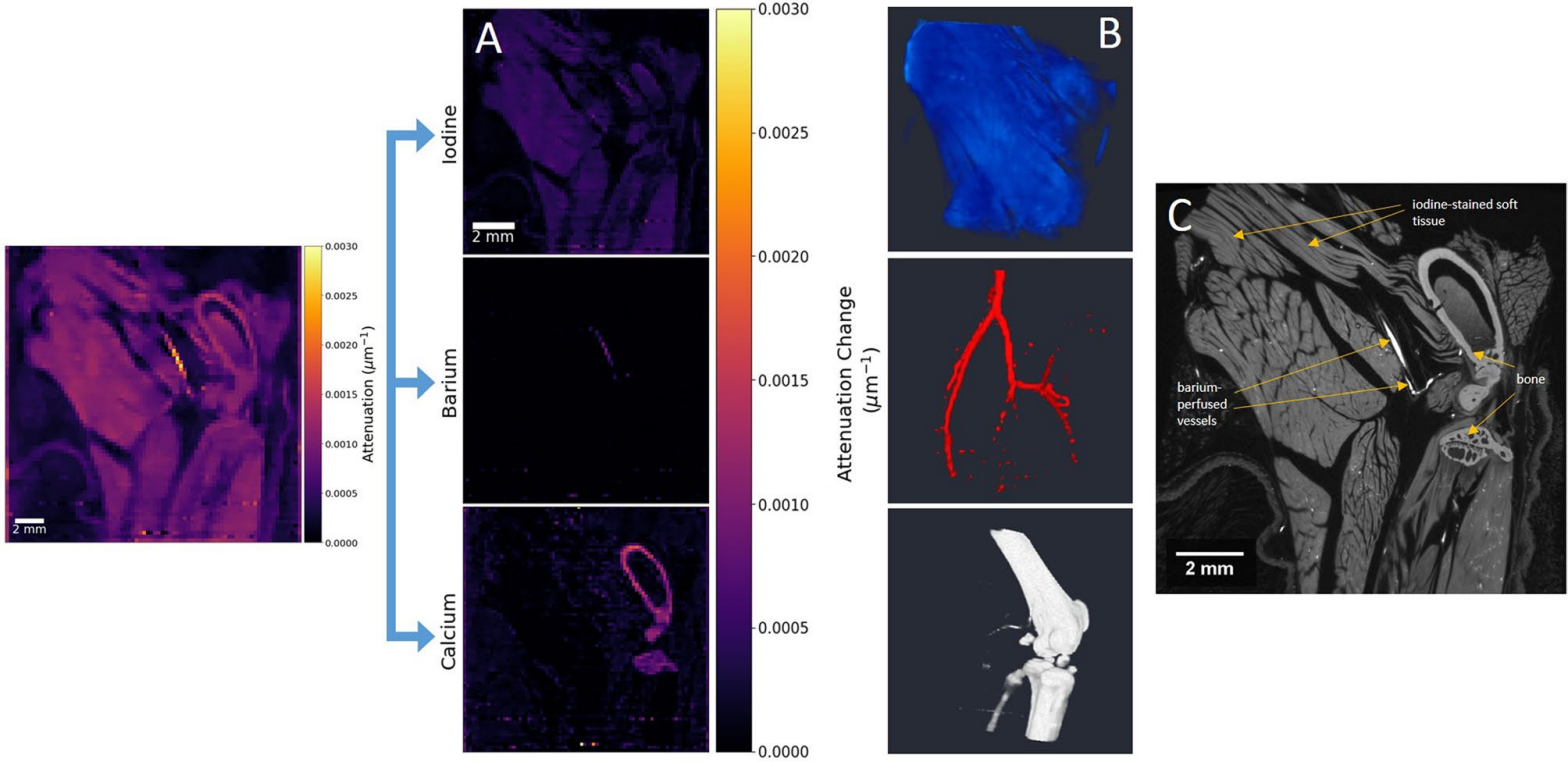
Elemental segmentation of hindlimb by K-edge subtraction. (**A**) Elemental difference maps shown for the hyperspectral image slice shown in the sagittal plane (left). Colour bar for elemental difference maps is measured in terms of attenuation change, Δµ, as determined by KES of narrow energy windows (5 channels, ≈ 1.1 keV width total) either side of each absorption edge. (**B**) 3D volume mapping for elemental maps of iodine-, barium- and calcium-containing regions (top, middle, bottom respectively). (**C**) Virtual cross-section from a high spatial resolution XCT scan of the mouse hindlimb, showing the equivalent image slice following volume registration. Labels indicate the three material phases with expected stain uptake regions.

In order to precisely map the distribution of chemical stain over soft tissue structures, the method of K-edge subtraction (KES) is applied. For each absorption edge, narrow channel ranges either side of the edge position are integrated and subtracted from one another. As a result, only information corresponding to the chosen element remains, enabling a form of elemental segmentation to be performed. Applying the KES method for iodine and barium produces the results shown in Fig. 3A, where we achieve clear segmentation of the iodine- and barium-stained structures. Included in Fig. 3B are a set of 3D volume renders for the segmented regions, where we also map the remaining material phase, following subtraction of iodine and barium (also see Supplementary Video 1). As expected, the structure of this phase over 3D is confirmed as calcium-containing bone. In order to reinforce the identification of each phase, an equivalent image slice acquired through conventional XCT for high spatial resolution comparison is shown in Fig. 3C. The scan was reconstructed with a voxel size of approximately 14 µm, providing clear structural insight which may be missing in the hyperspectral images. Exact image slices were identified and matched using volume registration. As seen in Fig. 3C, labelled regions for each stained structure matches those segmented using the KES method in the hyperspectral dataset. Based on the XCT image shown, we can identify the soft tissue regions stained by iodine, with the barium contrast agent only filling the lumen of blood vessels in the mouse hindlimb. Though a small amount of iodine binds to the bone tissue as previously discussed, such differences are negligible relative to the soft tissue, and do not emerge in the elemental difference maps created.

Our second sample, a triple-stained mouse forelimb, provides an opportunity to observe the effect of overlapping contrast agents. Here, we now have additional soft tissue staining provided through the tungsten-based phosphotungstic acid (PTA). While the use of PTA has been shown to provide strong contrast enhancement to regions such as connective tissue, it also enhances tissues overlapping with iodine-based agents, including muscles, while dual-staining in the skin is possible, though sometimes the skin is removed due to acting as a barrier for stain penetration^38^. Therefore the sample provides an opportunity to investigate stain interaction, and identify any double-stained regions or potential displacement of one chemical by another.

**Figure 4:**
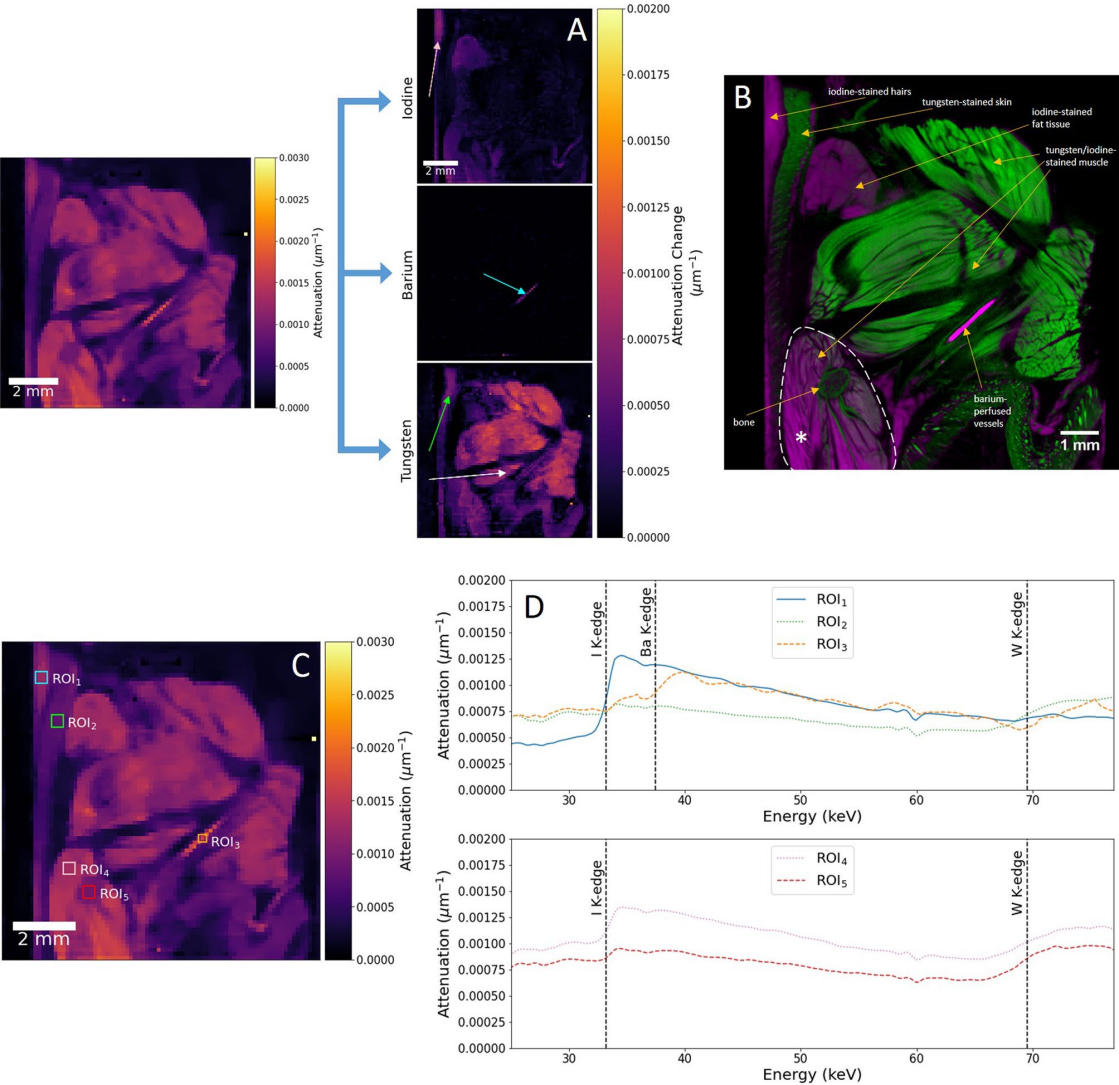
Spectral analysis for a triple-stained forelimb specimen. (**A**) Elemental difference maps shown for the hyperspectral image slice shown in the coronal plane (left). Colour bar for elemental difference maps is measured in terms of attenuation change, Δµ. Arrows are included to highlight distinct soft tissue regions stained by each chemical, confirmed as hair (pink), vasculature (blue), skin (green) and muscle (white). (**B**) Reconstructed cross-section from a high spatial resolution DECT scan of the mouse forelimb, following basis material decomposition. The image slice matches that shown in the hyperspectral dataset, following volume registration. Labels indicate regions containing tungsten and calcium (green), as well as those containing iodine and barium (magenta). DECT decomposition fails to fully segment all elements into distinct phases. The dashed region and asterisk highlight the area below the elbow joint where PTA did not fully stain. (**C**) Reconstructed cross-section slice in the coronal plane of the triple-stained mouse forelimb. ROIs are shown for five distinct regions of the specimen, to analyse average voxel spectra. All ROIs cover 3 × 3 pixels, apart from ROI_3_, which covers a 2 × 2 region. (**D**) Voxel spectra for the ROIs selected in (**C**). Known K-edge positions are overlaid, with ROIs 1, 2 and 3 matching the K-edges for iodine, barium and tungsten respectively (top). Two ROIs, 4 and 5, show the presence of both iodine and tungsten contrast agents (bottom).

Using the same reconstruction and analysis routine as for the double-stained specimen, we acquire a full spectral segmentation of the three contrast agents in the triple-stained mouse forelimb, as shown in Fig. 4A. It becomes immediately clear that PTA constitutes the dominant stain in the majority of the soft tissue, with significantly reduced iodine uptake compared to the double-stained hindlimb sample. Clear elemental segmentation is achieved despite the addition of a third elemental stain, owing to the unambiguous identification of each absorption edge with hyperspectral imaging, followed by KES for each spectral marker (also see Supplementary Video 2). By analysing an equivalent DECT reconstructed dataset of the same sample, we can perform a direct comparison of imaging modalities, while using the high spatial resolution nature of the DECT method to identify and match soft tissue regions. As with the XCT comparison for the double-stained specimen, we once more perform volume registration to directly match the volume orientation. The DECT image slice shown in Fig. 4B, with a reconstructed voxel size of approximately 8 µm, was segmented following basis material decomposition, a method of separating the individual contributions of each material to the overall attenuation of the X-ray beam^9^, and has regularly been used as an effective method of chemical segmentation in DECT^10,39^. The decomposition method for DECT is limited in its ability to fully segment each individual chemical phase. The higher attenuation tungsten-stained regions, along with the calcium-based bone, can be segmented from the iodine- and barium-stained regions, but no further separation is possible. In part, this is due to the aforementioned similar spectral profiles of iodine and barium, with very similar attenuation and closely-spaced K-edges. Hyperspectral CT can overcome such issues due to the high energy resolution available, enabling segmentation through KES. We can therefore confirm the identification of iodine staining in the hair of the forelimb, as well as the fat tissue, between which a layer of skin is clearly stained only by the tungsten-based PTA. The intensity of the PTA staining shows clearly in the majority of the musculature, with very low levels of iodine present. This is reversed in the lower area below the elbow joint, as highlighted by the dashed region in Fig. 4B, where iodine staining shows an equal or greater intensity than the tungsten contrast agent. This is due to the staining time of the PTA, combined with its slow penetration speed, meaning the contrast agent only partially stained to this sample depth. This did however offer an excellent opportunity to evaluate the balance of stains in the same soft tissue structure. Finally, a small section of bone is noted by the DECT results, which appears as part of the tungsten segmentation in the hyperspectral KES analysis. As such, no calcium map is extracted following KES. The cause of this may be investigated through the analysis of several ROI voxel spectra, as shown in Fig. 4C and D. The results confirm the presence of iodine, barium and tungsten in the hairs, vessels and skin respectively, based on reference regions in the DECT dataset. The spectra of ROI_4_, at the lower region of the forelimb, shows the overlap between I_2_KI and PTA staining in the muscle tissue. A small step change at the K-edge of tungsten is also observed in ROI_5_, taken over the feature identified as bone in the DECT cross-section. Therefore, partial binding of the PTA contrast agent to the bone tissue may have occurred, indicating why no calcium segmentation was possible. A slight rise in attenuation around the iodine K-edge was also present, though slightly offset from the known edge position. Combined with its small step size, the signal was likely due to noise, or possibly of sufficiently low concentration to not register as part of the iodine map in Fig. 4A.

### Quantifying chemical stain distribution

While the use of KES confirms the presence of each stain, and allows us to build unambiguous individual elemental maps, this form of spectral analysis enables elemental segmentation but provides no quantitative insight into the concentration distribution of the staining agents. The analysis may be taken a step further using 'step size' measurements, making use of the spectral markers available. By applying a fitting routine to the spectral profiles the height of the K-edge step may be extracted for every voxel. It has previously been shown that the height of the absorption edge step (referred to as Δµ_0_) is directly proportional to the concentration of the corresponding elemental species^16^. Therefore we can extract a map of Δµ_0_ values by performing this linear fitting routine across the entire reconstructed volume, as performed previously on a single-stained biological specimen^17^. However, on its own the use of this 'edge height' analysis can only provide relative concentration values, rather than absolute values. In order to extract a quantitative map of chemical staining, we require the use of calibration samples of known concentrations.

**Figure 5:**
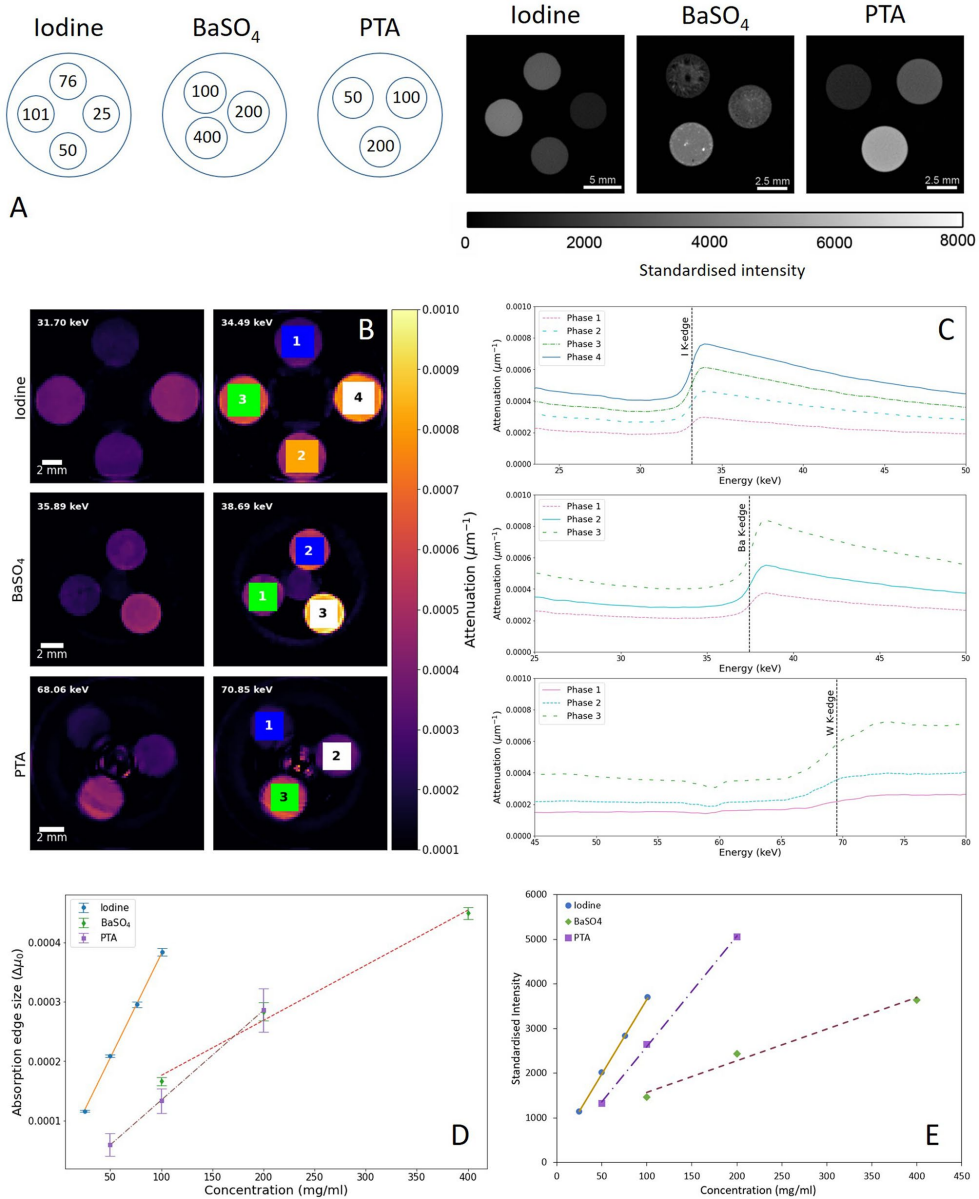
Design and spectral analysis of chemical phantoms. (**A**) Illustration of the phantom layout for each contrast agent (left), with chemical concentrations labelled in units of mg/ml. The I_2_KI phantom is labelled as iodine to highlight that the concentrations represent aqueous I_3_^−^. Reconstructed image slices for each of the respective chemical phantoms (right), with orientation matched to the concentrations labelled. The BaSO_4_ phantoms appear heterogeneous due to partial sinking and separation of the barium sulphate crystals from the agarose base. Images are measured in terms of 'standardised intensity' value, with the same overall scaling range. (**B**) Reconstructed slices of each chemical phantom, shown in the axial plane, for two energy channels, taken just before (left column) and just after (right column) the relevant K-edge position. ROIs covering each chemical phase are highlighted and numbered, from which spectral plots were extracted. Minor ring artefacts appear on the PTA reconstructed phantom. (**C**) Voxel spectra showing attenuation as a function of energy for the ROIs highlighted in (**B**). The attenuation values were averaged over the full ROI. Known K-edge positions of the phantom’s heavy element are overlaid. (**D**) Fitted relationship for each chemical phantom, based on the average K-edge step change over six vertical slices through the sample depth. Error bars measure the standard deviation across the slices analysed. A line of best fit is applied for each phantom following linear interpolation. (**E**) Fitted relationship between chemical concentration and 'standardised intensity’ value in each phantom phase following conventional XCT imaging. Values were calculated for the central slice reconstruction. Phantom datasets were scanned at 80 kV and 100 µA with a Zeiss Xradia MicroXCT-400. Standardised intensity values are based on 2% agarose and air, as the barium phantom uses an agarose base.

For this study, a set of three calibration phantoms were produced, containing various concentrations of the contrast agents used in our biological specimens: I_2_KI, BaSO_4_ and PTA. Figure 5A illustrates the layout of each phantom sample, and the chemical concentrations for each container. Also shown in Fig. 5A are reconstructed image slices for each phantom, acquired through conventional XCT. Signal intensity is measured in terms of 'standardised intensity', with higher values corresponding to a more strongly-attenuating (higher concentration) material. While typically CT number would be used to quantify signal intensity, we choose a different terminology to account for the fact that calibration was performed using a combination of agarose and air, as opposed to the standard water/air. The heterogeneous nature of the BaSO_4_ phantoms are due to partial sinking and separation of the barium sulphate crystals from the agarose base during mixing and cooling.

Reconstructed image slices of the chemical phantoms are shown in Fig. 5B. Image slices are evaluated at two different energy channels, taken either side of the K-edge positions for the heavy elements found in each phantom (iodine—33.17 keV, barium—37.44 keV, tungsten—69.53 keV). As expected, a rise in attenuation is observed across the appropriate energy threshold for the phases. The tungsten-based PTA phantom portrays some signal distortion due to minor ring artefacts. Spectral profiles are then extracted by selecting ROIs covering each distinct phase, and averaged to cancel out any differences due to chemical inhomogeneity. Figure 5C shows the ROI voxel spectra for each phase of the three phantoms. The profiles closely follow the expected trend of a sharp rise in attenuation at the K-edge position, with the overlaid edge energies demonstrating the precise matching with the measured experimental data. The step change at the K-edge varies in size for each phase, therefore a relationship between chemical concentration and absorption step size may be determined.

Using the aforementioned edge height analysis routine, the absorption edge size may be measured and plotted against the corresponding phase concentration. Further, by evaluating these values at a range of vertical heights through the sample depth, any changes due to inhomogeneity may be accounted for. For each phantom, a total of six slices through their vertical depth were measured and averaged. The fitted relationships are shown in Fig. 5D, with strong linear correlation found for each chemical phantom. Similar linear relationships have previously been identified between concentration and CT number^10^. A linear fitting was also confirmed between concentration and standardised intensity for the XCT acquisition of the phantoms in this study, and is shown as a direct comparison in Fig. 5E.

**Figure 6:**
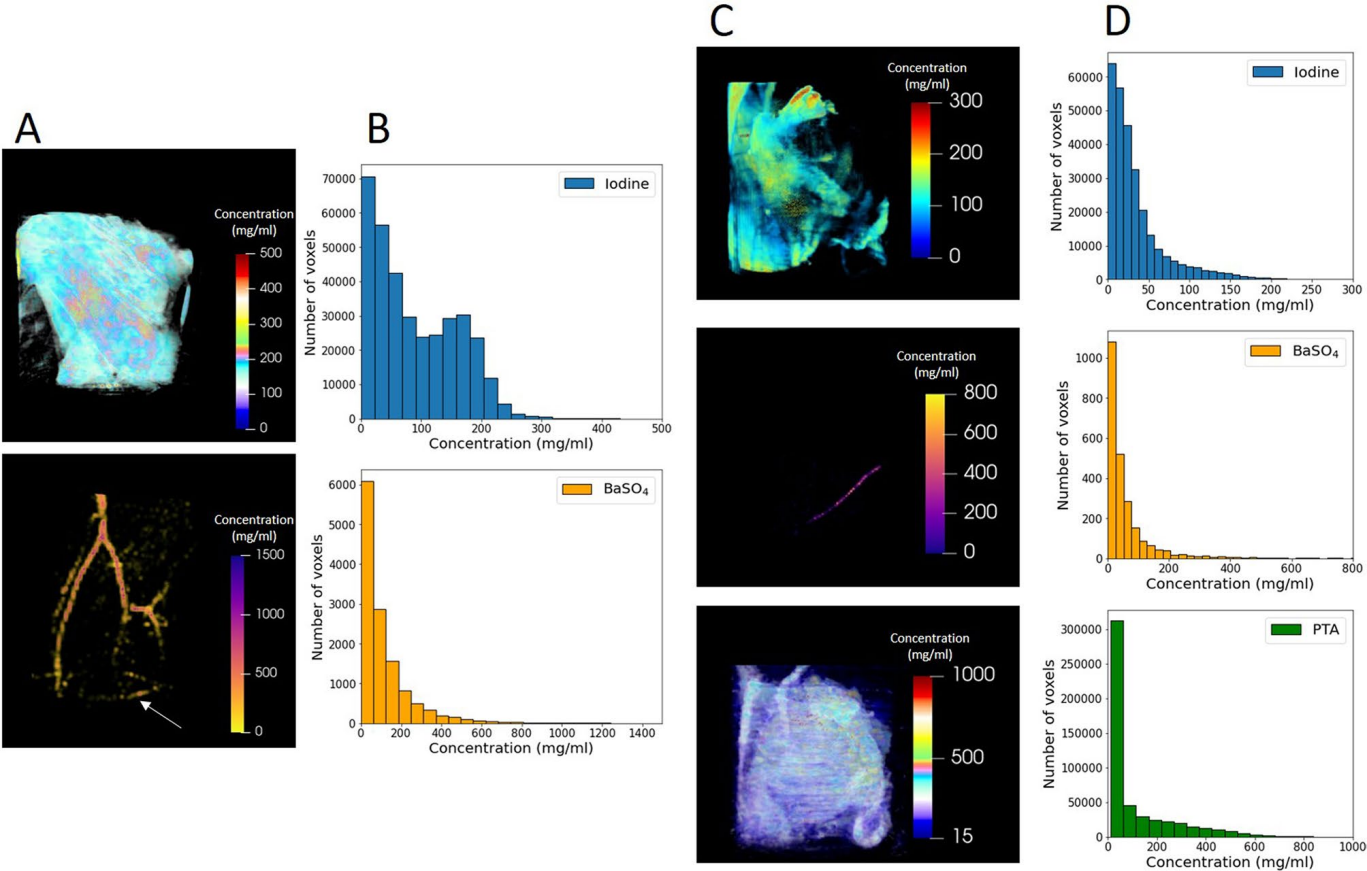
Distribution and concentration of contrast agents. (**A**) 3D volume maps quantifying the stain distribution of iodine (top) and barium (bottom) in the soft tissue and blood vessels respectively within the double-stained mouse hindlimb. Some low concentrations regions (white arrow) with a systematic pattern of voxels are attributed to ring artefacts or general noise. (**B**) Histogram detailing the statistical breakdown of concentration distribution on a voxel-by-voxel basis for each chemical stain of the sample in (**A**). (**C**) Volume maps for the triple-stained mouse forelimb, showing absolute concentration values in each voxel for iodine (top), BaSO_4_ (middle) and PTA (bottom). (**D**) Concentration distribution histogram for each stain within the sample in (**C**).

With a linear relationship determined for each contrast agent, a direct conversion from relative to absolute concentration may be calculated for every voxel in the biological specimens. As a result, stain distribution as a function of position can be investigated. The results for both the double- and triple-stained mouse limbs are shown in Fig. 6A and C, which illustrate 3D maps measured in terms of concentration on a voxel-by-voxel basis. Analysing the results in 3D provides excellent insight into the level of staining at various depths of the tissue (see Supplementary Videos 3–7). In addition, Fig. 6B and D detail the statistics behind the concentration distribution, with histograms showing the concentration frequency across the entire volume. Analysis of the iodine staining in the double-stained specimen shows nearly all voxels contain ≤ 300 mg/ml of iodine, with the highest concentrations observed on the exterior skin. For the vasculature, the largest vessels experience chemical uptake of up to 800 mg/ml of BaSO_4_. For many smaller vessels, concentrations of below 200 mg/ml were typically observed. However, consideration must be taken for the partial volume effect. Given the relatively coarse spatial resolution (151 µm) of the reconstructed volume, smaller vessels typically occupied a space below a single voxel. Therefore, the edge height analysis cannot precisely estimate the true concentration within such vessels. A number of voxels also portray a very low concentration of barium disconnected from the main vasculature structure. Some regions are attributed to noise and artefacts, though some cannot be ruled out as smaller regions of the vessel structure with sufficient barium signal to register on the chemical map. An improved spatial resolution would enable us to unambiguously identify and discriminate noisy regions from stained structures.

The concentration information details for the triple-stained forelimb sample are shown in Fig. 6C and D. These results show the significantly lower concentration of iodine observed throughout the various soft tissue regions. With a maximum recorded concentration of approximately 250 mg/ml, the majority of iodine-stained tissue falls below 100 mg/ml. Using the images in Figs. 4 and 6, we observe that the iodine staining shows clearly in regions where there was no tungsten uptake, such as the external hairs, and layers of fat within the specimen. As expected, barium was only located within the blood vessels, with uptake averaging 100 mg/ml, but reaching as high as 800 mg/ml for very few voxels in the largest regions of the vessel. Less of the vasculature was observable for the triple-stained specimen, believed to be due to a larger number of vessels below the spatial resolution limit. The more highly-attenuating tungsten-based PTA contrast agent dominated the soft tissue staining, as seen in the elemental segmentation. Connective tissue and muscle regions are predominantly enhanced by tungsten staining, though some overlap is observed in the musculature with both iodine and tungsten. Particularly in the lower section of the forelimb, where the muscle tissue was only partially stained by PTA, we see a clear presence of both the iodine- and tungsten-based contrast agents.

The statistical analysis also provides a metric of the detectability of the hyperspectral system, with many voxels measured to contain iodine and barium concentrations of approximately 1% (w/v) for both mouse limb samples (see Supplementary Figs. S3 and S4 for extended histogram plots). However, caution must be taken in evaluating the low concentration sensitivity, as highlighted by the PTA statistics, where a heavier skew towards the lower concentrations appears due to the presence of some ring artefacts remaining. The results of the calibration phantoms in Fig. 5 confirm that for an ideal, homogeneous sample, detection of K-edge markers is currently achievable down to concentrations of 2.5% (w/v), particularly when aided by iterative algorithms aimed at reducing noise fluctuations in the spectral domain.

## Discussion

Using a set of chemical calibration phantoms, evaluation of double- and triple-stained biological specimens was achieved in this study by hyperspectral X-ray tomography. The work builds on previous studies involving hyperspectral imaging^16,17^, exploiting the variety of spectral analyses available to achieve elemental segmentation over a full 3D volume. Due to the high energy resolution of the HEXITEC detector, unambiguous chemical identification is achievable for a range of elements in the hard X-ray range simply by matching of the spectral fingerprints (absorption edges) to their unique positions. With the ability to identify multiple chemicals simultaneously, and determine the absolute concentration distribution as a function of position, the work offers great potential for investigations involving heavy metal uptake, even in cases where we have no a priori knowledge of initial chemical composition. In the research of ecology and agriculture, heavy metal uptake by soil and vegetation is of particular interest. Excessive uptake by soil and plants of heavy metals, including molybdenum (Mo), cadmium (Cd), and lead (Pb), can pose a serious threat to animals and humans, due to their toxic nature when consumed in high concentrations^40–42^, on top of the damaging nature of such elements to vegetation yield and quality. Calculating similar calibration metrics for concentration of commonly-occurring elements in agriculture using hyperspectral tomography would enable future studies into uptake and distribution across soil, plant, animal and human samples to evaluate the level of contamination and poisoning risk. The feasibility of using hyperspectral imaging for such work requires further evaluation. X-ray CT as an imaging technique has an inherently low sensitivity, compared to alternative light-optical methods^43^. Therefore, given that biological systems typically exhibit heavy metal levels of parts-per-million^41^, hardware improvements in spectroscopic systems require continued advancement to become a viable option. Nevertheless, this research offers a reference point for a workflow in potential future studies. In addition, the present study analysing iodine and PTA staining provides a means of better understanding tissue morphology following heavy element accumulation. This work enables improved interpretation of the tissue layers in which these elements accumulate, providing a useful tool for future toxicological research.

This work is the first of its kind to establish a relationship between K-edge height and chemical concentration, and in theory is capable of defining a baseline calibration for each element, translatable across research studies. Given that each calibration phantom only includes a single high-attenuation element, and each energy channel is quasi-monochromatic, it is believed that the linear relationships between K-edge height and chemical concentration are independent of both sample and incident X-ray spectra. Attenuation response, regardless of concentration, is consistent over the same subset of energy channels, and therefore alterations to the spectra only correspond to a change in photon statistics. Therefore, any changes are proportional, and the same linear relationship will emerge. Future work may validate this approach through imaging of the same calibration phantom under different beam voltages. Potential sources of error may include differences in spectral responses for other hyperspectral detectors, as well as the choice of reconstruction algorithm, given that these can directly impact voxel attenuation spectra due to noise or excessive smoothing of the K-edge.

A feature of spectral analysis covered in this work that could warrant further investigation is the concept of 'spectral unmixing' for multi-element voxels. That is, in instances where a single voxel registers multiple absorption edge markers, signifying the presence of more than one chemical element. For hyperspectral imaging, this is more likely given the large pixel size and coarse spatial resolution of the HEXITEC system. As such, precise quantification of the concentration distribution for these 'mixed' voxels may be affected, particularly in cases where the absorption edges are closely-spaced. While many voxels will likely contain a single element, this study has already identified some cases of such mixing, such as the overlap of PTA and iodine in the muscle tissue (see Fig. 4). A range of unmixing methods have previously been explored in other fields of spectral imaging^44^, in order to better classify material composition and improve spatial resolution. The methods typically focus around linear unmixing routines, applied to fields such as energy-dispersive X-ray diffraction^45^ and fluorescence microscopy^46^, while more recently machine learning approaches have been studied for 'blind' spectral unmixing^47^. Such algorithms could easily be adapted to improve quantitative measurements in hyperspectral X-ray CT.

This research also highlights some of the existing limitations of hyperspectral imaging and the HEXITEC detector, the main aspect being the spatial resolution limits. With a pixel size of 250 µm and a 2 cm × 2 cm detection area, magnification of samples is limited, with typical spatial resolutions ~ 100 µm. As such, the current detector technology may require a complementary high-resolution technique, followed by volume registration, to achieve optimum spatial feature definition and composition mapping in the low-micron range. Similarly, sample size is currently limited by the available field of view. It is anticipated that this limitation will be overcome as more sensitive and more pixellated detectors emerge. At present, the modality is restricted to smaller specimens, or magnified ROI studies.

The sensitivity of the detector is also an area for further investigation. Here, concentration levels on the order of 10^1^–10^3^ mg/ml or more were studied. For studies involving trace elements, detectability levels of 10–100 × greater may be required. Further quantitative studies could be conducted on the HEXITEC sensitivity limit using a set of heavy metal phantoms, decreasing in concentration until spectral markers may no longer be observed. It seems likely that future developments in hyperspectral detectors will improve the sensitivity further. Alongside this, additional developments in denoising algorithms could extend the detection limit of trace elements yet beyond those currently achievable. It should also be noted that hyperspectral imaging offers the potential for trace analysis through other complementary imaging modalities. For example, the use of energy-dispersive XRF has been shown to offer successful elemental identification on the trace level, both for heavy metal elements, as well as those at the low-energy sensitivity limit of such detectors^48–50^. The effect of self-absorption often confines the XRF modality to studying small samples^51^, however this limit was already apparent given the HEXITEC pixel size and field of view (FOV), and thus the multi-modal approach is achievable for small samples with large features. Alternatively, a two-step approach may be adopted, whereby hyperspectral CT offers non-destructive identification of ROIs, followed by cutting and extraction of the ROI, which may then be investigated under high-resolution, high spectral sensitivity XRF to overcome self-absorption concerns.

## Conclusion

To conclude, we have evaluated the potential of hyperspectral X-ray tomography for the direct segmentation, measurement and mapping of absolute stain concentration. This research demonstrates the first instance of directly extracting elemental concentration from multi-stained biological specimens through evaluation of absorption edge height for several elemental markers. With an excellent energy resolution (~ 1 keV), hyperspectral CT overcomes the issues of spectrally similar attenuation profiles observed in DECT, shown here in the unambiguous discrimination between iodine- and barium-staining. Through a set of hindlimb and forelimb murine samples, the work has enabled a thorough examination of the interaction between multiple simultaneous chemical stains for double- and triple-staining procedures, providing insight into the level of stain overlap in the same soft tissue regions, and how this affects the overall concentration distribution. Evaluating the results against equivalent high-resolution XCT and DECT datasets, the advantages in the spectral domain become clear, yet the issues in the spatial domain are also highlighted, particularly with regards to the need for improved spatial resolution. However, through either the improvement in spectral detector technology, or the use of multi-modal acquisition for combined high spatiospectral resolution, the methodology opens up the possibility of chemical investigations in a range of fields. Given sufficient sensitivity, exploring heavy metal uptake in ecological or environmental research, as well as contrast agent applications for medical biopsies, would be greatly enhanced by the ability to confirm the elements present, and even more so by quantifying the concentrations and how they are distributed. As such, the studies conducted in this work show a further step in the vast potential for hyperspectral imaging moving forward.

## Supplementary Information

Supplementary Information 1.

Supplementary Information 2.

Supplementary Information 3.

Supplementary Information 4.

Supplementary Information 5.

Supplementary Information 6.

Supplementary Information 7.

Supplementary Information 8.

## Data Availability

All datasets used in this study are available via Zenodo, at https://doi.org/10.5281/zenodo.6787594 for the biological samples, and at https://doi.org/10.5281/zenodo.6787489 for the chemical phantoms.
