# Supplementary figures and images for: Quantifying multiple stain distributions in bioimaging by hyperspectral X-ray tomography
Source: Sci Rep. 2022 Dec 19;12:21945. doi: 10.1038/s41598-022-23592-0 (PMC9763266; doi:10.1038/s41598-022-23592-0)

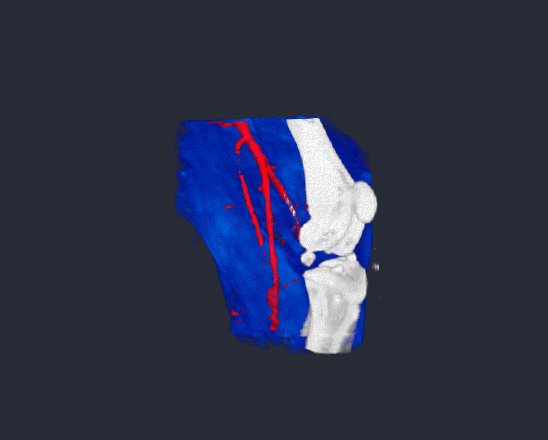

Supplement: Supplementary file 1 — Supplementary Information 1. [file 41598_2022_23592_MOESM1_ESM.gif]

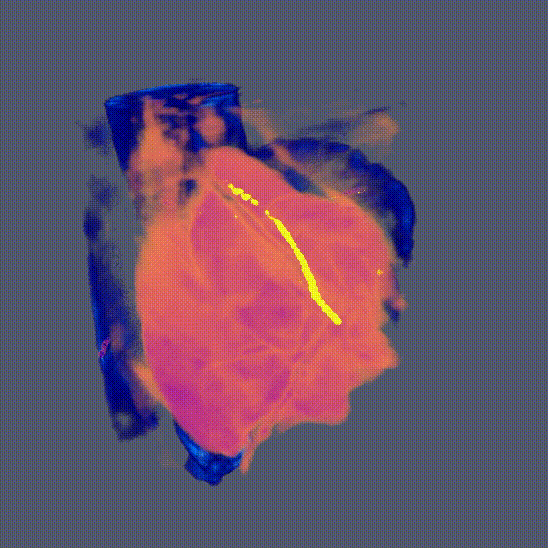

Supplement: Supplementary file 2 — Supplementary Information 2. [file 41598_2022_23592_MOESM2_ESM.gif]

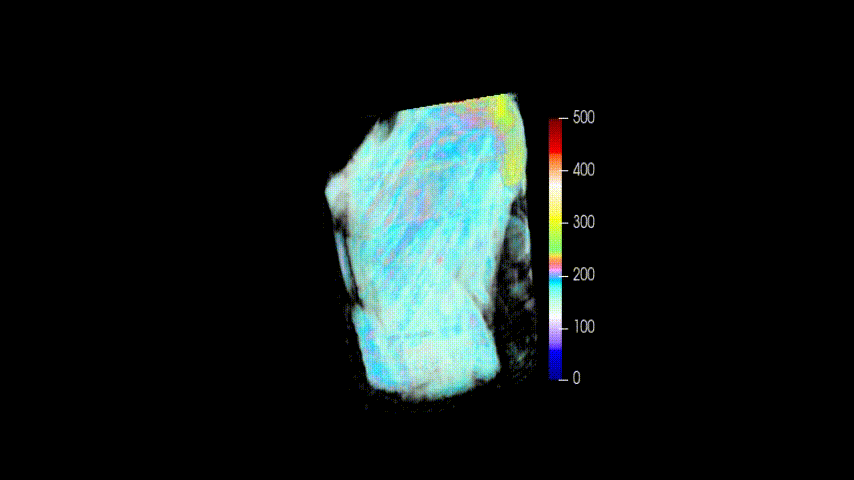

Supplement: Supplementary file 3 — Supplementary Information 3. [file 41598_2022_23592_MOESM3_ESM.gif]

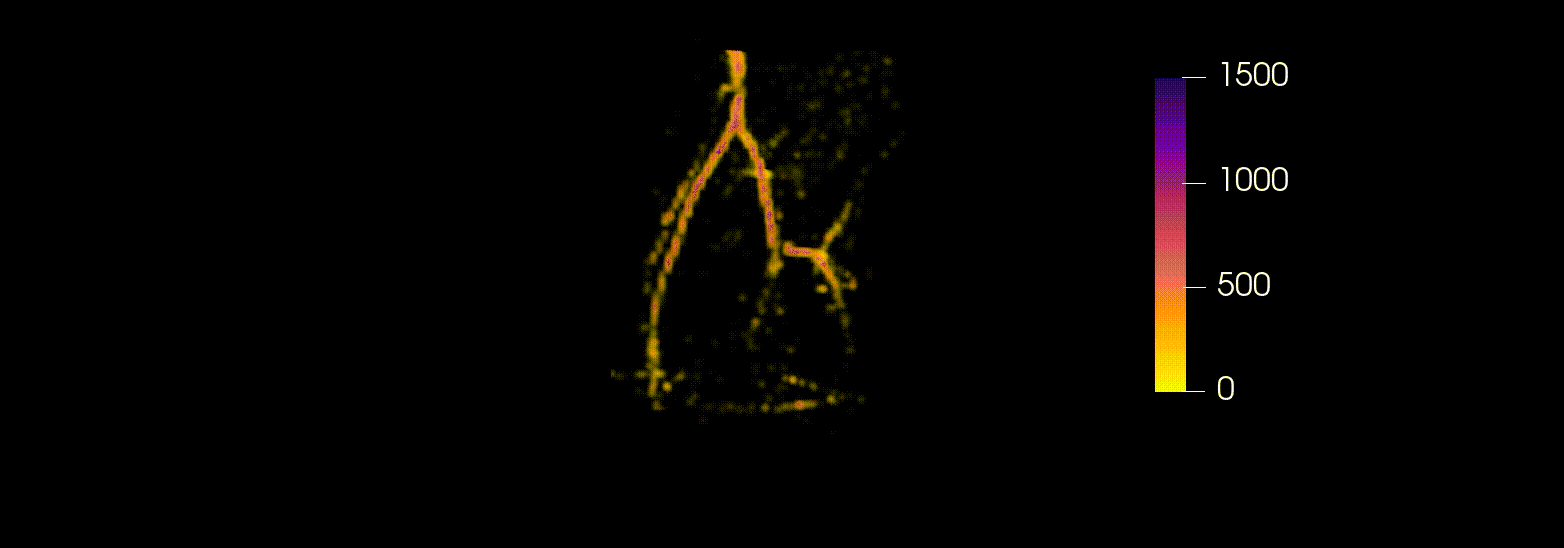

Supplement: Supplementary file 4 — Supplementary Information 4. [file 41598_2022_23592_MOESM4_ESM.gif]

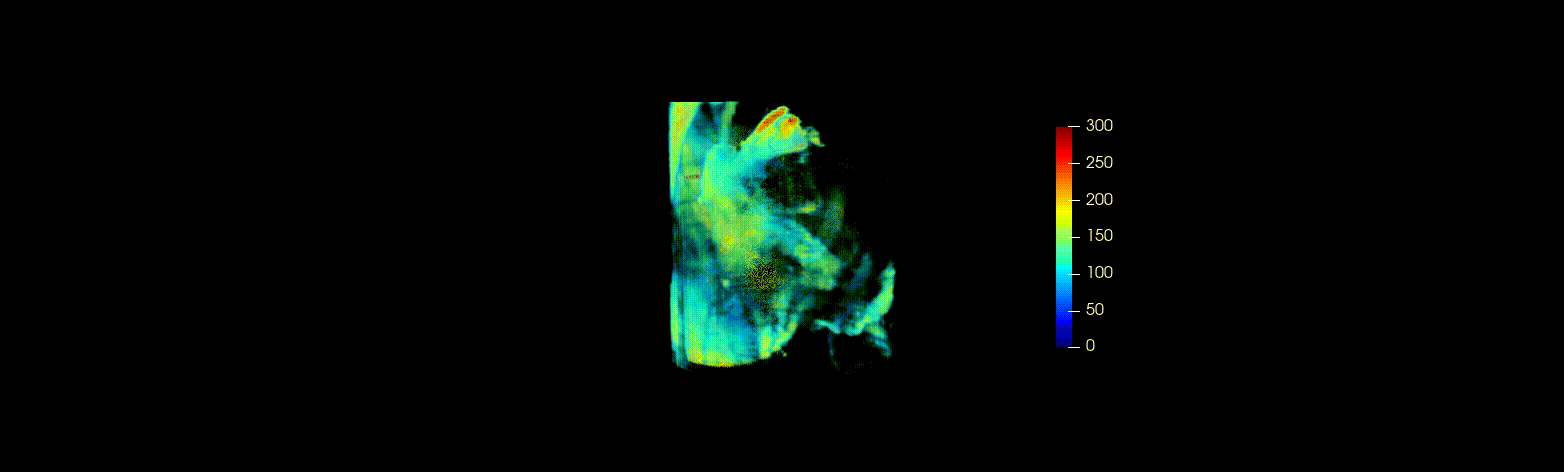

Supplement: Supplementary file 5 — Supplementary Information 5. [file 41598_2022_23592_MOESM5_ESM.gif]

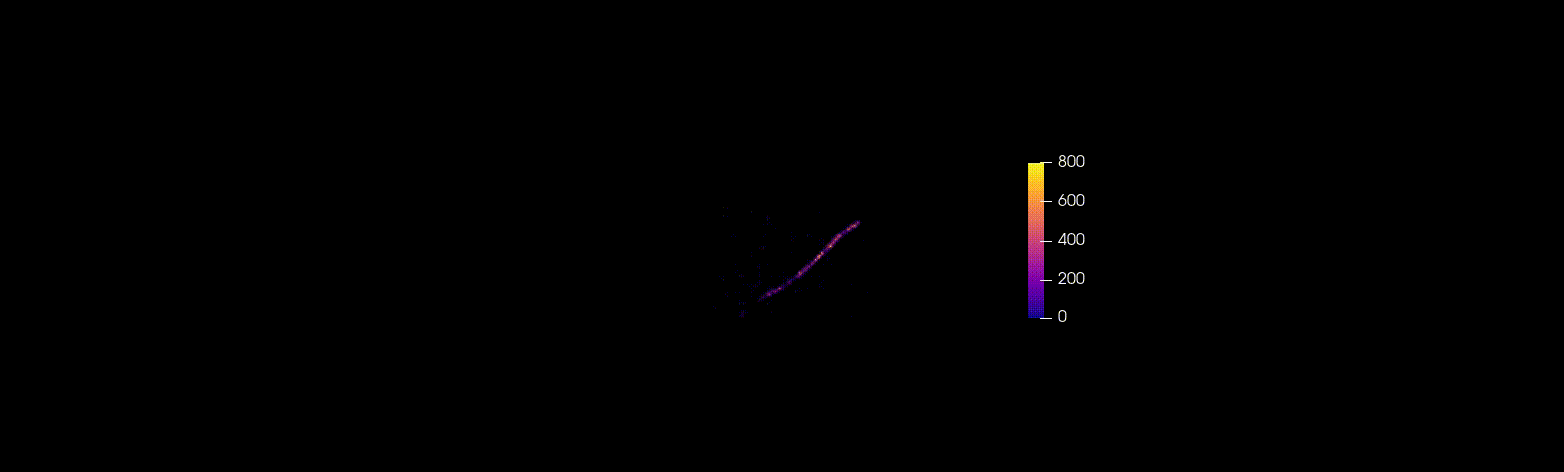

Supplement: Supplementary file 6 — Supplementary Information 6. [file 41598_2022_23592_MOESM6_ESM.gif]

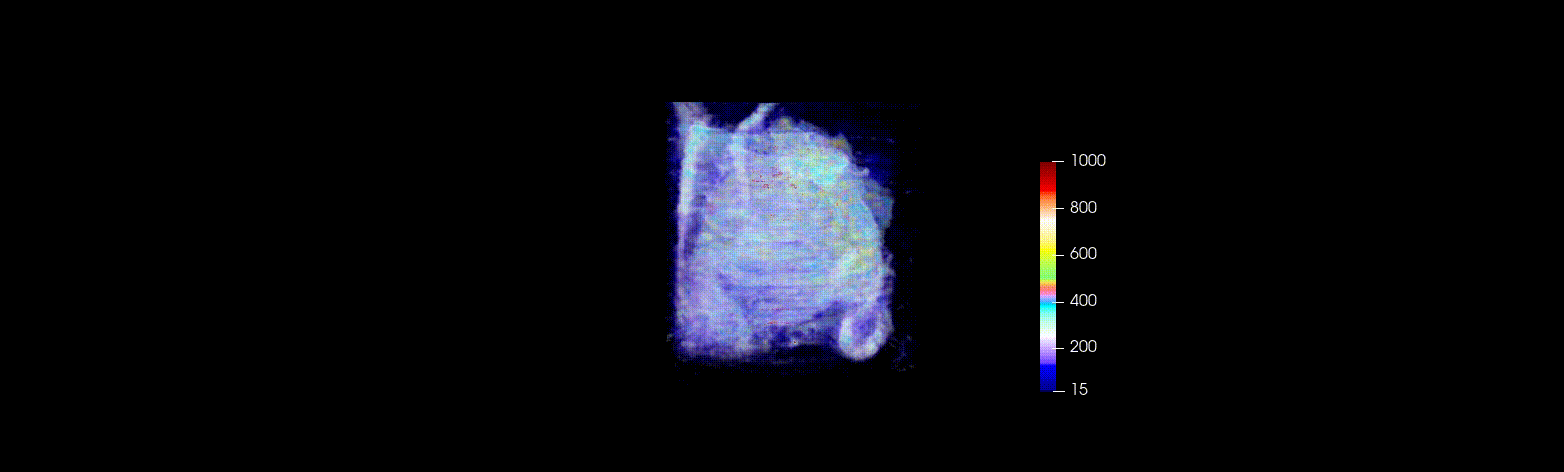

Supplement: Supplementary file 7 — Supplementary Information 7. [file 41598_2022_23592_MOESM7_ESM.gif]
