# Supplementary material for: Quantifying multiple stain distributions in bioimaging by hyperspectral X-ray tomography
Source: Sci Rep. 2022 Dec 19;12:21945. doi: 10.1038/s41598-022-23592-0 (PMC9763266; doi:10.1038/s41598-022-23592-0)
Supplement: Supplementary file 8 — Supplementary Information 8. [file 41598_2022_23592_MOESM8_ESM.docx]

**Supplementary Information**

Quantifying multiple stain distributions in bioimaging

by hyperspectral X-ray tomography

Ryan Warr^1,*^, Stephan Handschuh^2^, Martin Glösmann^2^, Robert J. Cernik^1^, and Philip J. Withers^1^

^1^Henry Royce Institute, Department of Materials, The University of Manchester, Manchester, M13 9PL, UK

^2^VetCore Facility for Research, University of Veterinary Medicine Vienna, Vienna, Austria

^*^Corresponding author – ryan.warr@postgrad.manchester.ac.uk

**Hyperspectral energy-channel calibration and resolution calculation**

By convention, hyperspectral detectors store incident photons into hundreds of narrow energy ‘bins’ or ‘channels’. Through a calibration using spectral markers of known energy, it was possible to calibrate the system, and establish a relationship between channel number, and photon energy. The calibration data consisted of a series of fluorescent X-ray peaks, emitted from metal foils (Ba and Tb) that were exposed to a radioactive ^241^Am source. Figure S1 shows the calibration dataset for a single pixel, with 5 peaks highlighted, corresponding to the K_α_ and K_β_ fluorescence peaks of Ba and Tb, as well as the ^241^Am photopeak. The channel number at which each peak occurred was determined using a peak-finding algorithm. The energy values of the peaks are known and well-established, and therefore by plotting channel number against energy (in keV), a relationship may be determined. Also shown in Fig. S1 is the linear fitting of the relationship, based on the 5 data points collected. An *R^2^* value of 0.99 for the fit confirms a strong linear relationship, with a polynomial fitting giving the equation of the fit as:

Energy (keV) = 0.278 x Channel Number + 1.23


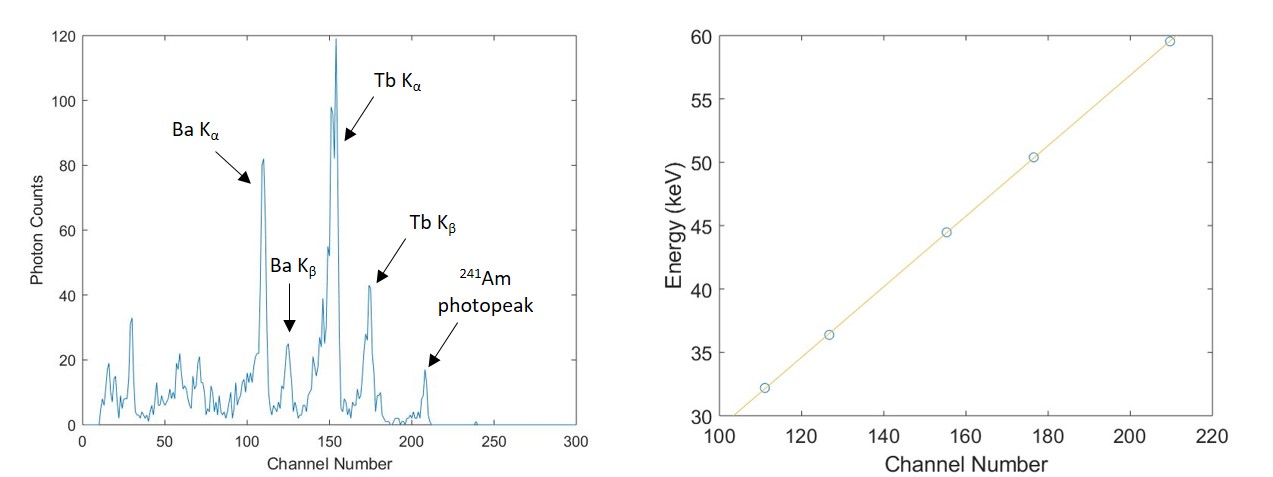


**Supplementary Figure S1: Energy calibration using the ^241^Am radioactive source.** (Left) Single pixel (40, 40) plot of the calibration spectra, measuring photon counts as a function of channel number. The highest energy peak at 59.5 keV ^241^Am photopeak is labelled, as well as the XRF peaks produced from Ba and Tb metal foils upon exposure to the ^241^Am source. (Right) Matching of the channel number for each XRF peak and the ^241^Am photopeak to their known energy values, enabling a linear relationship between channel number and energy to be determined.

To determine the energy resolution of the system, the same calibration dataset was used. By measuring the FWHM of the ^241^Am peak for every pixel, the resolution of the system was calculated. Figure S2 shows the frequency distribution of FWHM values measured over every pixel. Dead pixels identified during pre-processing were not included in the plot. Also shown is a pixel map over the full HEXITEC array, showing the distribution of FWHM values. Here, dead pixels were set to a value of 0. Based on the average value of functioning pixels, the resolution of the detector at 59.5 keV was measured to be 1.27 ± 0.47 keV.


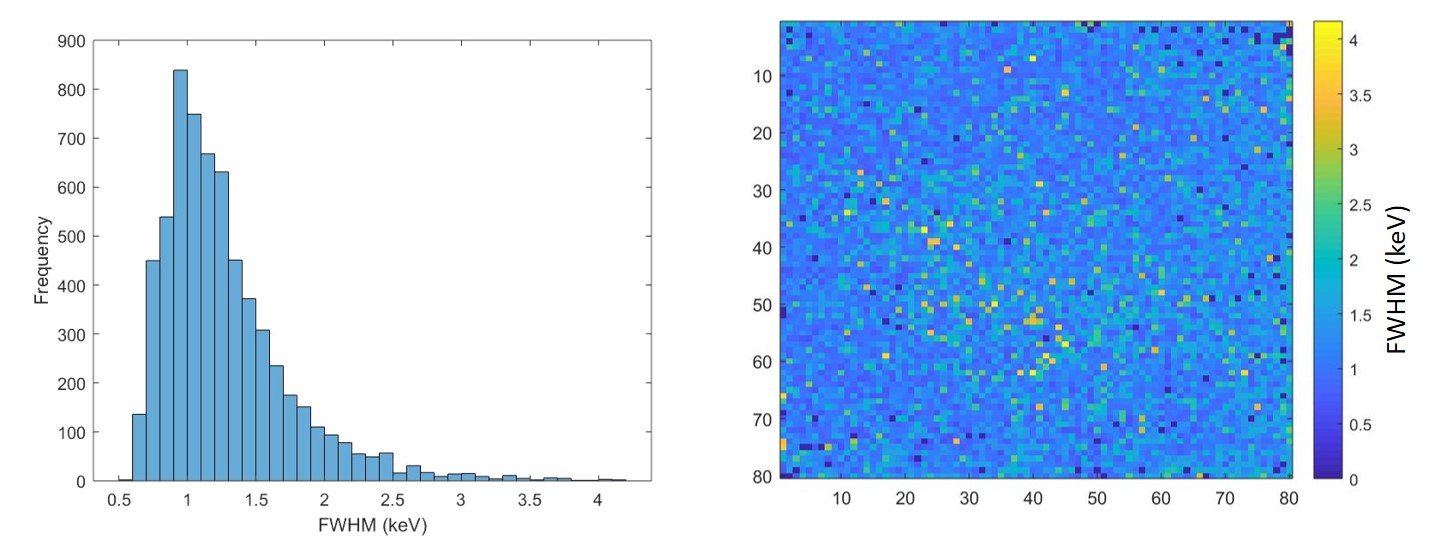


**Supplementary Figure S2: Measurement of the resolution statistics using the ^241^Am photopeak.** (Left) Histogram of the frequency distribution for FWHM values measured at 59.5 keV based on the ^241^Am photopeak, over the full detector pixel array. Dead pixels were not included. (Right) Pixel map of the FWHM distribution. Dead pixels were set to a value of zero.

**Considerations on HEXITEC low concentration sensitivity**

In order to better evaluate the voxel frequency of concentrations at the low value range, Supplementary Fig. S3 and S4 show extended histogram statistic plots, with a greater number of bins to reduce the average bin size, therefore providing a clearer pattern of concentration distribution. For the double-stained specimen (Supplementary Fig. S3), the iodine bin size was around 4.5, while it was 12.4 for the BaSO_4_ histogram.

Two clear trends are observed, with both iodine and BaSO_4_ distribution gradually descending in voxel number as concentration is increased. The iodine distribution also exhibits a small peak around 180 mg/ml, suggesting a particular region of soft tissue with a consistent concentration of contrast agent. The results indicate that, while noise fluctuations may cause some low value voxels, the trends do not indicate any outliers, and therefore we may assume that our detectability limit lies in this low concentration range, at approximately 5-10 mg/ml, equivalent to a concentration of 0.5-1% (w/v).

For the triple-stained specimen (Supplementary Fig. S4), however, this may be brought into question. 150 bins were used for the iodine and BaSO_4_ distributions, reflecting the reduced total range of concentrations compared to the hindlimb sample. This gives a bin size of approximately 3.2 and 8.5 for iodine and BaSO_4_ respectively. The PTA plot uses 250 bins as it covers a larger range, giving a bin size of roughly 10.2. While sensible trends are observed once more for iodine and BaSO_4_, a very sharp rise is observed for the lowest concentration bin for the PTA distribution, covering concentrations up to around 10 mg/ml (around 1% w/v). We attribute this to the presence of ring artefacts, distorting the voxel spectra and affecting the measured values of concentration. Therefore it is important we do not assume the detectability limit is as low as 1%, and caution must be taken when accounting for the effects of noise and artefacts. Given the results in the main manuscript for the calibration phantoms, concentrations of 2.5% and above appear much more reasonable as a lower limit estimate of sensitivity for the HEXITEC.

**Supplementary Figure S3: Extended concentration statistics for double-stained mouse hindlimb specimen.** Histogram statistics are shown, detailing the concentration distribution of iodine (top) and BaSO_4_ (bottom) over all voxels in the reconstructed volume. A total of 250 histogram bins are used over the full concentration range for both plots.


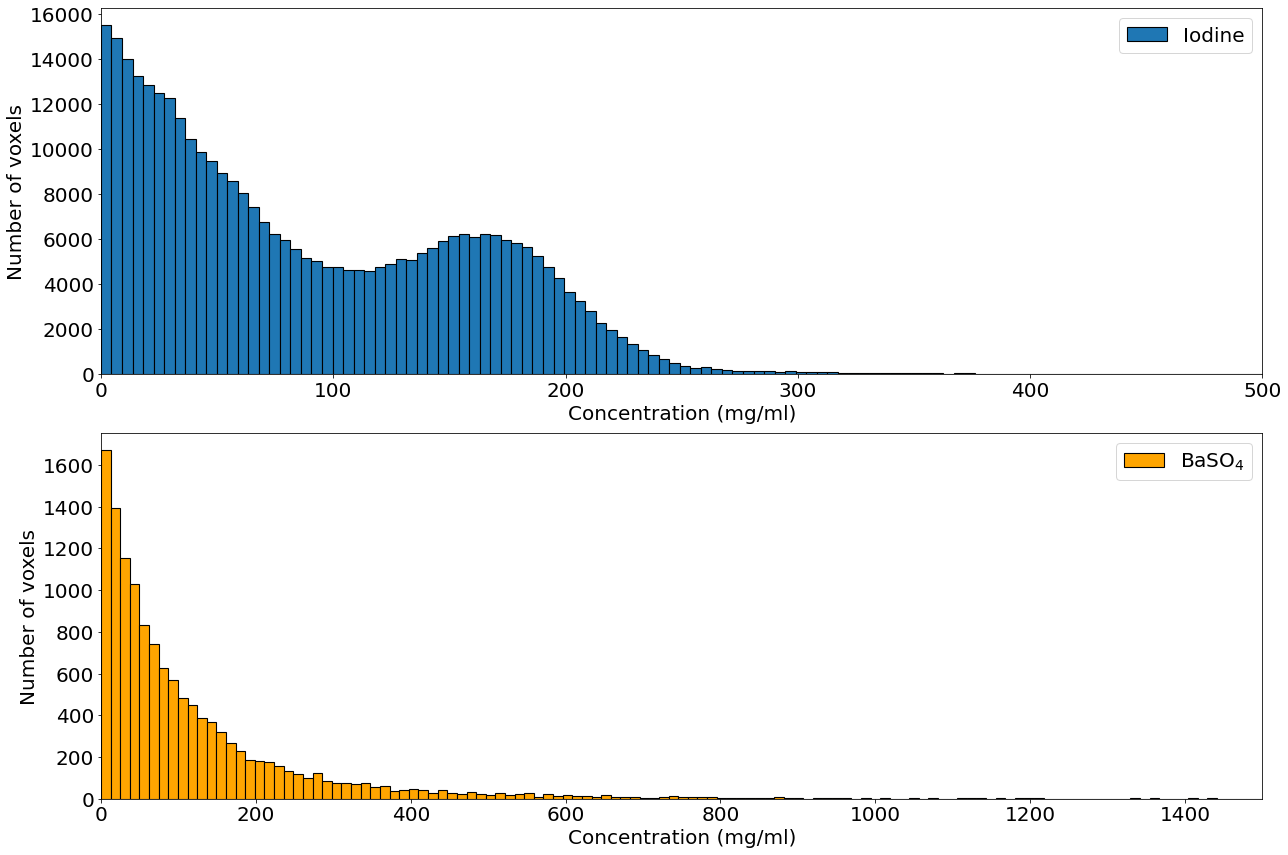

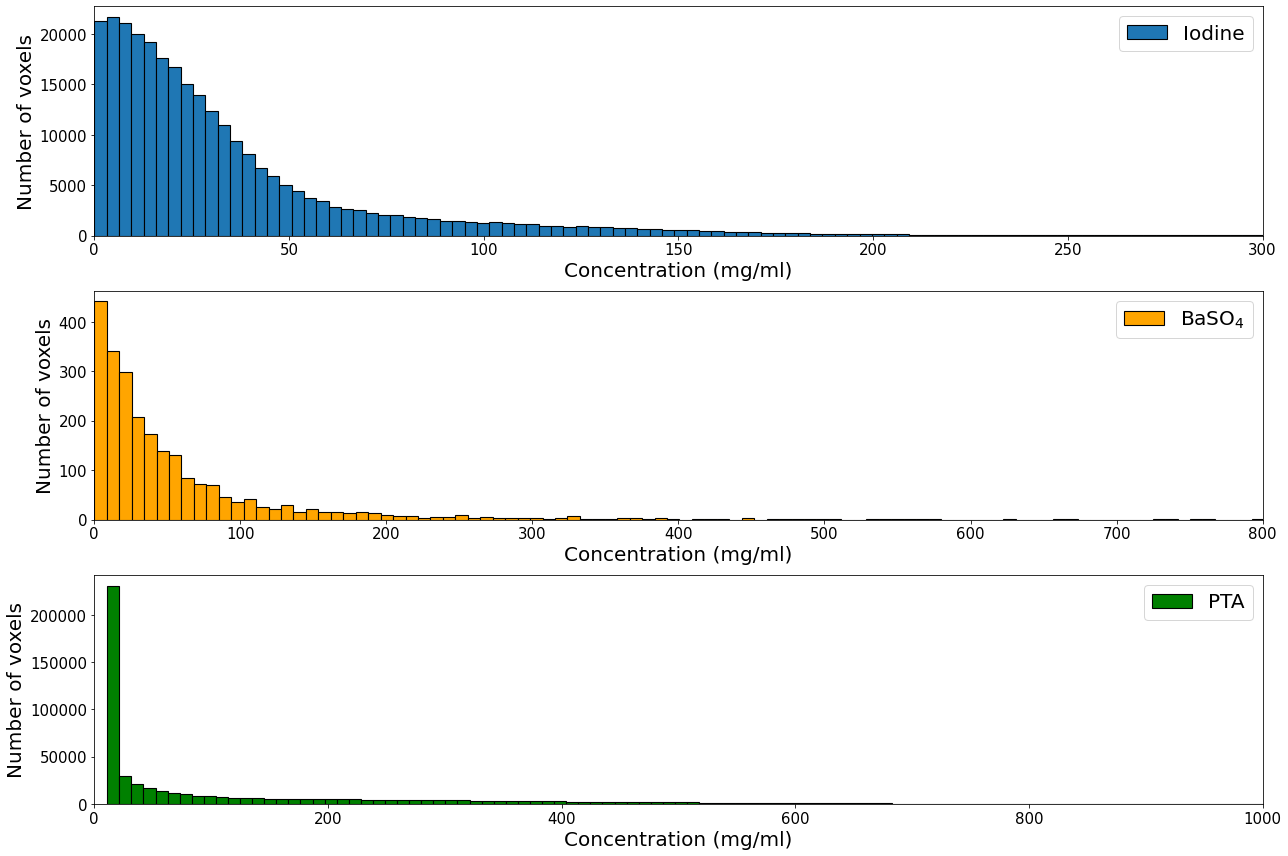


**Supplementary Figure S4: Extended concentration statistics for triple-stained mouse forelimb specimen.** Histogram statistics are shown, detailing the concentration distribution of iodine (top), BaSO_4_ (middle) and PTA (bottom) over all voxels in the reconstructed volume. A total of 150 histogram bins are used over the full concentration range for iodine and BaSO_4_, while 250 bins are used for PTA. The sharp increase in voxel frequency for the lowest concentration bin in the PTA plot is attributed to the effect of ring artefacts within the final reconstructed volume.

**Supplementary Video 1:**

K-edge subtraction mapping of the three phases identified in the double-stained hindlimb specimen. The segmented regions correspond to iodine-stained soft tissue (blue), barium-stained blood vessels (red), and the calcium-containing bone (white).

**Supplementary Video 2:**

K-edge subtraction mapping of the three phases identified in the triple-stained forelimb specimen. The segmented regions correspond to iodine-stained regions (dark blue), barium-stained blood vessels (yellow), and the tungsten-stained regions (orange).

**Supplementary Video 3:**

3D GIF of absolute concentration mapping of iodine within the hindlimb specimen. Colour bar measures concentration in units of mg/ml.

**Supplementary Video 4:**

3D GIF of absolute concentration mapping of BaSO_4_ within the hindlimb specimen. Colour bar measures concentration in units of mg/ml.

**Supplementary Video 5:**

3D GIF of absolute concentration mapping of iodine within the forelimb specimen. Colour bar measures concentration in units of mg/ml.

**Supplementary Video 6:**

3D GIF of absolute concentration mapping of BaSO_4_ within the forelimb specimen. Colour bar measures concentration in units of mg/ml.

**Supplementary Video 7:**

3D GIF of absolute concentration mapping of PTA within the forelimb specimen. Colour bar measures concentration in units of mg/ml.
